# An ISM-MICMAC-based study for identification and classification of preventable safety risk mitigation factors in mass housing projects following a BIM approach

**DOI:** 10.1016/j.heliyon.2024.e38240

**Published:** 2024-09-21

**Authors:** Amir Mohammad Maleki Toulabi, Towhid Pourrostam, Babak Aminnejad

**Affiliations:** a Department of Civil Engineering, Qeshm Branch, Islamic Azad University, Qeshm, Iran; b Department of Civil Engineering, Central Tehran Branch, Islamic Azad University, Tehran, Iran; c Department of Civil Engineering, Roudehen Branch, Islamic Azad University, Roudehen, Iran

**Keywords:** Safety hazards, Urban constructions, Mass housing projects, BIM, ISM-MICMAC

## Abstract

Construction operation is among the most high-risk sectors in terms of work-related accident, making it highly challenging to surveil the safety of such projects. In construction projects, failure to observe safety represents a leading cause of fatal accidents, not to mention the losses incurred by such accidents to national assets of the country. Accordingly, recent decades have witnessed the emergence of modern techniques for improving the occupational safety of construction projects. The main purpose of the present research is to identify and classify different preventable risk mitigation factors in mass housing projects following a building information modeling (BIM) approach. The research methodology included interviews with relevant experts and elites followed by analysis of the data on the 12 identified-as-significant variables for mitigating the preventable risk factors in mass house construction projects by means of the inferential – structural modeling (ISM) in MICMAC software. In order to explore the relationships among and succession of different criteria and further classify them at different levels, ISM was implemented, with the MICMAC software used to analyze the direct and indirect influences, develop influence/dependence maps, and judge about the role of each criterion. Findings of the present research showed that the mutual relations (H3), the reward system (H6), the reporting system (H7), and the supervisors' supervision (H8) are autonomous variables and hence impose the smallest contributions to the system. Accordingly, they can be eliminated from the model though their effects may not be completely ignored. On the other hand, the employees’ empowering (H4), the safety management system (H5), the teamwork (H9), the self-efficiency (H10), and the knowledge and awareness (H11) were identified as the linkage variables that fill in the gap between the safety and occupational accident reduction in the mass house construction projects. Further, the continuous improvement (H2) and the safe behavior (H12) were identified as dependent variables, implying that they exhibit the weakest influence coupled with highest dependence on any change in the conditions of the system. Last but not the least, the management commitment (H1) was identified as the only dependent variable which deserves lots of attention. This information can be helpful to safety decision-makers, end users, research organizations, and academic institutes who work to reduce the preventable risk factors in mass house construction projects.

## Highlights

• ISM-MICMAC can be used to handle the complex interaction problems in real world.
• The model addresses dependent relationships between the various criteria.
• The results can provide the best way to improve existing safty processes.

## 1 Introduction

Each year, millions of work-related accidents occur around the world. Some of these accidents are fatal while some others lead to temporary disabilities that may take several months to recover [[1], [2], [3]]. Work-related accidents cause human suffering coupled with economic losses, imposing sever damages to the society [4,5]. Accordingly, prevention from such accidents has been acknowledged as a fundamental and prominent task. In many cases, accidents are a result of unsecure and/or unsafe conditions [6]. As of present, work-related accidents represent the third cause of death in the world and have been introduced as serious health, social, and economic risk factors in the developed and developing societies [7]. The World Health Organization (WHO) has referred to the work-related accidents as an epidemic in the public health. The jobs in the construction industry comprise a remarkable contribution to the world economy as they make up some 7 % of the jobs around the world; in the meantime, due to the hazardous nature of the worksite and associated safety issues, the construction works alone are responsible for 30–40 % of the injuries/fatalities around the globe [[8], [9], [10]]. It has been estimated that some 100,000 labors die in construction sites each year (i.e., 1 death every 5 min), which is generally attributed to inappropriate and/or illegal working conditions. This fact indicates the importance of researching in the field of construction safety [11].

Following the mines, construction workshops host the largest number of and most sever work-related accidents, as compared to other works. According to stats, 30 % of the work-related accidents in Iran occur in the construction industry, and the rate of fatal work-related accidents has been estimated at 15 % in this industry [12]. Every year, some 270,000,000 work-related accidents are reported, through which some 2,200,000 labors die [13]. Unsafe conditions in the construction sites are usually a result of unsecure operation by workers or application of inappropriate tools and equipment in hazardous environments [14]. The most common work-related accidents faced by workers in construction sites include falling from a height, falling objects, falling debris and excavation wall, falling lift, electrocution, etc. Investigations have shown that some 80 % of the accidents occurring in construction sites, which cause either injury or death, can be prevented by observing basic safety measures at negligible cost. Examples of such measures include the installation of temporary edge protection barriers to avoid falling, securing material lifts, compulsory use of personal protection equipment (PPE, including safety helmet, safety shoes, and safety belt), and observing safety distances in the vicinity of high-voltage power lines [15]. A major problem encountered in the field of safety management is its disintegration from construction management [16]. With the safety planning being disintegrated from the operation plan of the project and in absence of associated links, it is highly difficult for safety managers to analyze what safety measures shall be taken at which place and which time [17], making such an effort highly complicated and inefficient [18]. In this case, risk identification is ineffective as two-dimensional maps demonstrate the building components rather than the construction process. Therefore, it is impossible to identify all risks before the construction is accomplished [19]. Safety engineers and experts shall utilize their personal experiences to identify the safety risks and then determine the required pieces of equipment, and such a manual and barely experience-based approach is susceptible to errors caused by personal judgments made by the decision-maker [7]. This implies that construction safety management can be improved by developing new techniques that can address the problems of the traditional approaches [20]. At the other end of the spectrum, it is necessary to institutionalize the safe thinking via culture-building among the involved individuals, including the top management, workshop management, engineers, workers, and supervisors, which is though to strengthen the efficiency of safety plans [21].

In general, preventable risk factors in construction projects can be classified under either of four categories, namely (1) political and strategic factors, (2) workflow-associated factors, (3) managerial factors, and (4) human-related factors, with these four categories being interconnected to one another [25]. Table 1 describes each category briefly.

**Table 1:** Factors affecting the safety of construction projects.

Factors	Description
**Political and strategic**	Safety policies and regulations strongly affect the safety of civil projects. Relevant laws constitute a framework by which the health and safety are controlled and regulated. All project managers must follow these regulations and laws and penalties shall be considered for possible violations [25]. Not only weak organizational culture of safety and inefficient definition of safety responsibilities and safety protocols, but also lack of proper safety policies end up with poor safety performance of construction projects. The regulations and their enforcement largely contribute to the safety of construction. Accordingly, safety regulations shall be seriously considered when designing job activities and setting safety policies of the company [26].
**Workflow-associated**	The workflow refers to the process of doing the jobs by construction workers, which may render harmful to their health and/or safety. There are organizations where jobs and tasks are done by systematically exposing the worker to hazardous activities. The main point in relation to the workflow-related factors is to pay attention to efficient control over the usually large number of subcontractors in a construction project. Indeed, the wide diversity of activities required in a construction project calls for a variety of subcontractors to get the job done [23]. As the number of subcontractors increases, so does the risk of work-related accidents because of the increased risk of miscommunication or efficient control. Occasionally, a contractor may transfer the entire deal of their responsibilities to subcontractors without ensuring that they are capable of providing a safe work place [25,26].
**Managerial**	This factor refers to the management's safety-related behaviors and orientations. This set of safety-related behaviors and orientations manifest the safety culture of the management. The safety culture of an organization is determined by the management's commitment to safety and its promotion throughout the organization. The management's commitment to safety has been identified as determinant factor controlling different types of communication and information exchange to all levels of a construction project [25].
**Human-related**	This factor refers to human aspects of construction activities. It considers the workers' safety behaviors and orientation, partly reflecting the safety culture of the organization. The safety culture is a subset of the organizational culture which encompasses the safety and health-related beliefs and values. A way of examining the safety culture is to research the workers' attitudes toward safety. This can shed light on the contrasts between workers' attitudes and evaluate the efficiency of safety programs. Accidents may arise from wrong behaviors or poor safety attitudes among the workers, which are very difficult to surveil and control [25,26].

### 1.1 Literature review

Accurate identification of potential safety risks is of paramount importance to the safety planning process. Presently, safety planning for construction projects is accomplished independently of the operation plan for the entire project, involving numerous players. This independence and the resultant disconnections cause particular problems for safety engineers, making it difficult to analyze what safety measures shall be taken at which place and which time to prevent possible risks. This industry hence needs an improvement to inefficiencies of paper-based and manual safety processes at worksites [22]. Today, various tools have been developed to integrate the risk analysis into the management process as a necessary and inevitable step in the construction industry. As an example, building information modeling (BIM) has demonstrated its extensive yet unique potentials in the construction safety management. By digitally representing different characteristics of the building, this technology facilitates the decision-making process for project managers, contractors, and various stakeholders in different phases of the project [23].

A mass housing project refers to the construction of multiple standard houses in one or more than one geographical location but under one master project plan, single management, and the same contract. The UN Economic Commissions recommends the construction of 10 houses per year for every 1000 individuals in developing countries, as a requirement for addressing the present and future needs for housing. By definition, we considered a project “mass house construction” if it involved constructing and delivering at least 10 residential units on the same premise [24].

The building information modeling system has been defined as a concept for describing the computer-facilitated instrumentation, processes, and technologies used to inspect building information, performance, planning, construction, and, finally, operation. It is highly effective for solving problems related to excessive changes in different stages of project management, from design to construction, operation, and cost and time management. As a powerful tool, BIM can contribute to continuous improvement of the design and implementation of mass house construction projects [25]. BIM introduces the details of the 3D/2D models, with their unique characteristics, into 2D drawings and relevant specifications. The most outstanding property of the BIM lies in the fact that each and any member designed in BIM comes with not only a multi-dimensional physical identity but also a set of information related to the designers' and managers’ activities and tasks. This information set covers the entire life cycle of the project from the early stages down to its completion, including the feasibility study to conceptual design, first- and second-stage studies, preparation, construction, installation and startup, operation, and even deconstruction. Briefly speaking, BIM refers to the process of generating and managing the building information throughout its life cycle. It establishes an intelligent linkage among different design elements and virtually enables the study of various scenarios for all groups of stakeholders. In addition, other design groups can see the effects of proposed modifications to their model on the project productivity. Finally, contractors can design a variety of successions, like the construction sequence, while they are virtually experiencing the design and development of the building model, performance, construction, and installation. In the construction industry, BIM-oriented design is among the best approaches to building modeling for realizing sustainable building design objectives, making it an ever-emerging topic around the globe. Thanks to its synergetic effect using optimization methods in the construction industry, BIM can open new horizons in the contexts of improved productivity, safety, shortened operation time, and decreased costs over the entire life cycle of a building [26]. BIM serves as a shared knowledge base for the building information, which makes it a reliable basis for decision-making during the life of the project from the beginning to the deconstruction stage [27]. A distinctive, intelligent, and virtual model is mainly provided by BIM to cover various design process aspects, comprising visualization, spatial conflict control, automated production and fabrication of parts, construction succession, and research/testing. All those involved in construction work, including architects, engineering consultants, contractors, and subcontractors, can then share this model [28]. The BIM technology shall be seen as an ideal application of product information in the construction industry, where the product is a building. The data bank can include a wide range of information about the product, including geometrical characteristics, material, construction and fabrication techniques, tolerances, costs, and even supply chain management information, either entirely or partially. Outputs of BIM help stakeholders all the way from the early ideation in the design stage to the construction phase and finally the operation stage [29].

The BIM-based safety management system can identify the complicated dynamic nature of the project automatically and present solutions or representations of protection systems in a responsive or automated way to reduce the identified risk factors [30]. Such a platform can further serve as a tool to present easily understandable up-to-date representations of the construction progress and safety throughout time, making it especially useful for identifying the hazardous locations in the construction site. Representation of the required safety actions to safety managers help them enrich their safety planning both in the early construction planning phase and during the construction phase. This can encompass planning for safer job routines and monitoring the execution of the planned routines in the construction phase [31]. Another significant capability of a BIM-based model is the development of an automated system for implementing the existing safety rules and procedures, best planning practices, and safety management. As the project proceeds, the model invokes automatically upon emergence of any hazard or unsafe condition, so as to avoid possible risks [32]. In addition, parametric BIM-based models can facilitate the assessment of the design-for-safety (DfS) knowledge against the safety rules, if any, so as to identify any possible construction safety issue in the design phase [31,33].

Before anything else, safe construction requires careful planning and monitoring of safety management through the entire lifecycle of the project (from design to construction planning, construction, and then extended to maintenance phase). Indeed, good safety actions and records provide for a positive, risk-free, and generative workspace. Pre-commissioning safety management planning is not only the first but also a fundamental step for the actual safety management [34]. Failure to identify a risk or hazard is usually a result of limited expertise or control by safety engineers or workers in the planning or implementation phase of the risk actions, not to mention the effect of poor training to construction workers. Each and any of these causes may end up exposing the workers to the risks of construction workspace. Safety planning usually encompasses probable risk identification and decision-making about relevant safety actions [35]. Important advantages of observing the safety management principles for construction workers include increased motivation to work, reduced work-related pressure, decreased risks of work-related accidents, injuries, and losses, elevated level of comfort, improved health and welfare, enhanced level of occupational health, etc. [11]. In addition to the workers, employers may receive such benefits as reduced probability of accidents, improved product quality and employee's efficiency, reduced rate of mistakes, decreased volume of rework, lower cost for health and medical treatment of otherwise injured workers, better utilization of the human resources, reduced production cost, etc. Considering the mentioned benefits, managers, decision-makers, and employers need, as soon and extensively as possible, to recognize the importance and value of the industrial protection and become aware of possible losses that may incur by industrial accidents (either directly or indirectly). Even more important is the value of the engaged humans and their health. The management needs to influence all workers and the work environment, identify the safety risk factors that can be mitigated to reduce the risk of work-related accidents, and make their best endeavor to protect the health and safety of the workers and the machineries in the best possible way [36].

Zhang et al. (2015) integrated safety regulations for fall-related risk identification and prevention into the BIM [31]. Hongling et al. (2016) combined BIM with safety design regulations to automate probable risk factors in the design stage [23]. Hossain et al. (2018) developed a risk assessment system that was integrated with the BIM to help architects and structural engineers evaluate different design components [37].

Lee et al. (2020) proposed an automation system for risk prioritization based on BIM and a revision system of design for safety. They predicted that the introduction of the concept of design for safety can minimize the construction-associated risks at construction sites by safety management in the design stage. Although numerous studies have focused on fall risk identification and automated BIM-based risk identification but they suffer from the limited scope of the search for construction risk identification. Lee et al. (2020) addressed this limitation by extracting a BIM-based risk prioritization scenario based on the worst-case scenario for automating the BIM-risk prioritization, on which basis they developed a risk assessment system [38].

Fargnoli and Lombardi (2020) reviewed the trends of research on BIM applications for improving workplace safety in construction activities during the last decade. Their systematic review highlighted knowledge-focused solutions, BIM-centered safety design, and dynamic visualization and feedback as the most suitable and promising areas for further research. The research findings revealed the essential practical role of 10.13039/501100024792BIM in supporting safety management, particularly ensuring a safer and more flexible atmosphere while enhancing quantitative risk analysis. Overall, a comprehensive review is presented in this study to add to the available knowledge of BIM-based tools for construction safety, contributing as a reference framework that enhances worker safety through the application of this technological innovation [39].

Wang et al. (2021) undertook fire risk assessment for building operation and maintenance activities based on the BIM technology. They used the so-called fire risk analysis for engineering to propose an indexing system for building assessment during the operation and maintenance periods at either of three levels, namely probable risk level, acceptable risk level, and secured risk level. In addition, they built a risk value calculation model for the operation and maintenance periods and codified a risk valuation standard for improving the firefighting ability of buildings. The results of this study showed that the BIM can be effectively applied to ensure that fire risk prevention and control capabilities have a more objective knowledge basis [40].

Pan and Zhang (2021) developed a new method for integrated BIM-based construction safety assessment in the design stage of construction projects. This methodology was composed of three indices, namely probability, consequence, and event. These indices were calculated by using objective and accurate data on occupational damages, fatalities, and construction planning. This research further included a case study to demonstrate the feasibility and efficiency of the proposed methodology [41].

Collinge et al. (2022) presented a BIM-based digital tool in the form of a safety risk library to help designers contribute to safety and health in digital environments. Considering the industrial need for knowledge sharing and collaboration, the proposed BIM-based safety risk library followed the Prevention through Design (PtD) approach, where safety risks are linked to corresponding remedies via various risk scenarios. Seeking to follow up the process of non-optimal safety and health management, this study utilized a conceptual framework that had roots in the construction guide. The data structure was implemented with the help of a 7-stage ideological methodology for improving the designer's knowledge of problems and access to such an expanding library (BIM-based safety risk library) [42].

Pourrostam et al. (2023) investigated the effect of BIM capabilities performance in construction projects by using FAHP and FTOPSIS. Results showed that consideration of BIM capabilities and application of hybrid methods of FAHP and FTOPSIS by construction project managers can acknowledge and enhance lean construction performance while mitigating work-related risks in such projects [43].

### 1.2 Problem definition

Despite all developments, the number of accidents and the resultant damages in construction projects are too high, and most of the existing safety risk identification and prevention frameworks are based on independent tasks and activities. In the recent past, the application of information systems and information and communication technology (ITC)-based modeling in the construction industry has expanded significantly, by which the practitioners have managed to significantly improve the success of construction projects in terms of both the final product and the general course of the project lifetime. Although such new modeling approaches have long been used in various fields, yet practical application of modern technologies for safety management in the construction industry is limited to the last two decades. One of such methods is the BIM, where the construction operation is simulated and designed virtually to help planners and safety managers. A review of the relevant literature demonstrates that the BIM can serve as a useful tool for safety risk prevention. Today, BIM is recognized as an important instrument for construction safety risk prevention. A major cause of implementing the BIM in safety management is its unique capability for preventing accidents and hazardous activities in the worksite.

### 1.3 Novelty of the research

Looking at history, it is evident that the construction industry has long been among the most hazardous industries worldwide, involving numerous accidents that led to fatalities and injuries. Construction work-related accidents cause significant harm to lives, fiscal assets, the environment, and corporate credit in civil activities every year. Researchers have expressed various opinions about the root causes of work-related hazards. These causes can be categorized under four broad classes: improper management, unsafe working conditions, inappropriate working conditions, personal factors, and job-related factors. In recent decades, many strategies have been proposed for identifying and preventing safety risks, the adoption of which has improved construction safety management. Alongside these laws and regulations, modeling has served as a useful tool for controlling the mentioned hazards, greatly helping relevant experts. The modeling does not require actual implementation and testing, implying that it can be quick, inexpensive, and highly repeatable to ensure proper results are achieved. Accordingly, application of modern technologies and knowledge to improve safety conditions and reduce work-related risks in construction projects can partly address the mentioned deficiencies in the construction industry. Various studies have used different methods to investigate the importance of safety in construction projects. However, an accurate piece of research focusing on the identification and prioritization of preventable safety risks in mass housing projects using the BIM-based ISM-MICMAC method is yet to be reported. The novelty of the present research lies in the fact that it considers the BIM approach and uses the ISM-MICMAC method to develop a model for the identification and prioritization of preventable safety risks in mass housing projects, so as to fill in the mentioned research gap. On this basis, building on the foundations and principles of the BIM, this work presents an attempt to identify and prioritize preventable safety risks in mass housing projects based on 12 criteria and their corresponding weights. The problem is analyzed by the ISM-MICMAC technique. For this purpose, the determination of the criteria and their weights were conducted with the help of safety experts in mass housing and academics.

### 1.4 Research hypotheses

The followings are the basic hypotheses considered in this research:

• The ISM-MICMAC technique can be used to classify the identified key risk factors and present a model of the associations among different risk factors to recognize preventable safety risks in mass housing projects based on BIM.
• Identification, updating, and investigation of the most important factors can contribute to the mitigation of work-related hazards in mass housing projects.

## 2 Materials and methods

### 2.1 Research method

This section presents the method of searching, summarizing, and analyzing the literature on the identification of the most important preventable safety risks in mass housing projects based on BIM. We undertook a systematic literature review of academic papers on preventable safety risks in mass housing projects based on BIM. To this end, the so-called nominal group technique (NGT) was employed to extract an exhaustive list of risk factors. The list was then given to a group of mass housing safety experts as well as several academics. Based on the feedback from the experts and the academics, a final list of the most important risk factors was obtained. The following subsections explain different stages of the research methodology in detail.

Stage 1: literature review

For the purpose of this study, we referred to credible scientific databases, including Emerald, Springer, Elsevier, Taylor and Francis, Wiley, etc … where we searched for articles, books, and master's and doctoral theses related to the preventable safety risks in mass housing projects, BIM, and ISM-MICMAC. The following search string was adopted:

Safety hazards; Risk factors; Uncertainty; Risk management; Mitigation factors; Strategy formulation; Preventable safety risk; Mass housing; Projects construction; Urban constructions; BIM; Pareto analysis; Interpretive structural model; Micmac and process optimization.

We considered only peer-reviewed articles in English language from scholarly construction engineering and management journals and did not put a time constrain as we are aware that some of the early papers on safety back to the early 1970s. There was a great degree of overlapping among the selected databases. Upon omitting the literature that violated the inclusion criteria or was of low quality, we ended up with a total of 174 research works for further study. Out of these, 82 pieces of work were evaluated using the quality assessment technique, which is an explicit systematic approach to the identification, selection, and critical assessment of research works [44].

Stage 2: NGT

NGT is a powerful tool for the soft system approach when developing ideas and reaching an agreement [45,46]. By encouraging mostly silent participants to participate and hence minimizing the effect of dominance on members, NGT ensures that every single individual has a chance to express his/her opinion and vote for any subject. To achieve the best results, it has been recommended to have 5 to 9 persons participating in brainstorming and NGT-powered structured decision-making sessions [47].

**Fig. 1:**
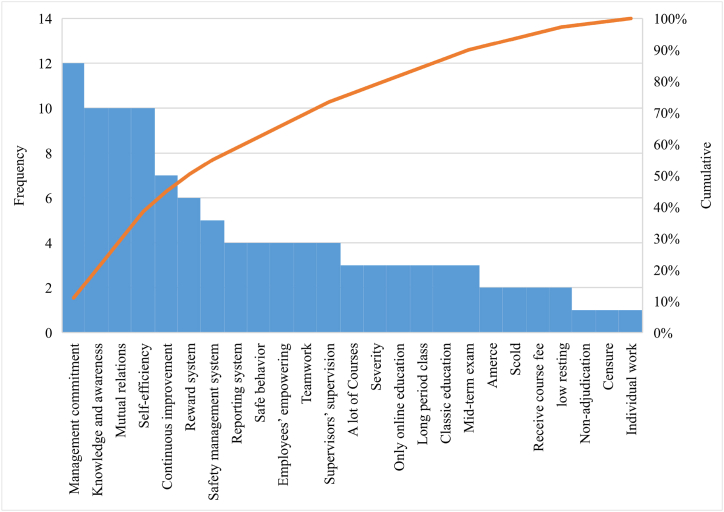
Pareto chart.

The Pareto principle in health and safety assumes that 20 % of the risks cause 80 % of the accidents, which implies that mitigation of these risks can largely reduce the probability of accidents. Although the cost of mitigating these risks can be high but it can cut the accident-related costs by as high as 80 %. On the other hand, application of the nominal group technique (NGT), which has been recommended by world health organization (WHO) for health and social research in 2014 [48], can offer even more accurate results. In the present research, following the BMI approach, 8 elites in the field of mass house construction safety and a couple of faculty members with more than 5 years of relevant experience were asked to identify the most significant preventable safety risk factors in mass house construction projects out of a comprehensive list of 55 factors. Their demographic details are presented in Table 2. This led to a list of 25 most significant factors, which where then reduced to 12 factors based on expert discussions and upon application of the Pareto diagram, as shown in Fig. 1. This set of 12 factors was used to develop the final Pareto diagram and identify the factors that contribute to 80 % of safety risk in mass house construction projects based on the BMI approach. Our findings can motivate practitioners and companies with activities in the field of mass house construction toward investing in the mitigation of the identified risk factors more seriously.

• *Factors affecting preventable risk mitigation in mass housing projects based on BIM*

**Table 2:** Demographic details of experts.

**Demographic details**	**Category**	**No of Experts**
Age	27–37	2
	37–57	4
	57–65	2
Experience	8–10 years	3
	10–15 years	2
	Greater than 15 years	3
Education	Postgraduate	3
	Ph.D.	5
Gender Male	From academics	5
	From industry	3
Female	From academics	6
	From industry	2

Although cause analysis of accidents and historical data provide valuable yet pretty general information for safety planning and management, this information are less than enough for predicting possible time and location of accidents in specific construction projects. The design and planning phase provide a vital opportunity for mitigating the hazards and accidents before they actually occur in the worksite. Existing approaches to the safety planning are usually text-based tools that work with various check lists on the paper or software spreadsheets. Inefficiency of existing methods of safety data processing and interpretation for safety decision-making in construction projects has been reported previously [49]. Earlier research has shown lack of responsive tools and resources for designers who seek to plan for construction safety. This has been while advanced technologies can potentially serve a key role in reducing the rate of work-related accidents, positively contributing to the existing routines in safety planning. During the recent decades, in an attempt to evolve the conventional safety management practices toward modern approaches to the project safety, such advanced information technologies as building information modeling (BIM) have developed into a widely accepted instrument in the architecture, engineering, and construction (AEC) industry [41].

Table 3 lists the main indices affecting preventable risk mitigation in mass housing projects based on BIM. In this research, these are extracted through library studies coupled with feedback from experts.

Stage 3: Implementation of ISM & MICMAC

**Table 3:** Factors affecting preventable risk mitigation in mass housing projects based on BIM.

Criterion	Sub-criteria	Coded sub-criteria	Description	References
**Organizational Level**	Management commitment (MC)	H1	Management commitment to safety is a key factor contributing to the safety atmosphere and indicates the supportive role of top management in safety programs at an organizational level. With an adequate commitment to safety, managers allocate sufficient resources to safety activities and strongly support those activities. A high level of management commitment affects the safety behaviors exhibited by employees.	[[50], [51], [52], [53], [54], [55], [56], [57], [58], [59], [60], [61], [62], [63]]
	Continuous improvement (CI)	H2	Continuous improvement (CI) can be defined as a planned, continuous, systematic process for increasing the organization's safety performance. The objective of CI in safety is the constant evolution of existing routines to realize better-than-before performance.	[[64], [65], [66], [67]]
	Mutual relations (MR)	H3	Mutual relations play a crucial role in achieving organizational goals and facilitating communications among employees, supervisors, and managers.	[[68], [69], [70], [71]]
	Employees' empowering (EE)	H4	Employees' empowerment (EE) refers to the ability of employees to observe safety measures. This ability usually develops by participating in decision-making sessions and safety meetings. As a critical element of the safety atmosphere, EE motivates employees to admit safety-related responsibilities and reduces high-risk behaviors by employees.	[[72], [73], [74], [75], [76], [77]]
**Safety Management Level**	Safety management system (SMS)	H5	Safety management system (SMS) refers to the methodologies used to link the organization to its safety policies, provide the required knowledge, and, finally, promote safety methods. Elements of an SMS include safety training courses and HSE maneuvers, to name a few. An SMS is further utilized to set macro safety policies and routines. This system deals with recognizing, assessing, controlling, and resolving safety issues across the organization.	[[78], [79], [80]]
	Reward system (RwS)	H6	The reward system (RwS) is a key factor contributing to the effectiveness of an SMS. It refers to methods by which top management of the organization encourages safe practices and inhibits hazardous practices	[[81], [82], [83], [84]]
	Reporting system (RpS)	H7	The reporting system (RpS) expresses employees' tendency toward reporting work-related safety issues. As an effective feedback loop, this system enables management to recognize safety hazards in the worksite properly, making it an organizational alarm system for safety. Providing a platform for submitting and storing employees' reports, RpS serves as a tool for learning lessons from previously occurred work-related accidents and hence prevent similar accidents in the future.	[[85], [86], [87], [88], [89], [90], [91]]
**Collective Work**	Supervisors' supervision (SS)	H8	Supervisors' supervision refers to their efforts to provide employees with safety training and surveillance. Studies have shown that employees may enjoy higher levels of safety performance if their supervisors spend more time surveilling their safety performance.	[[92], [93], [94], [95], [96], [97], [98]]
	Teamwork (TW)	H9	Teamwork measures communication, coordination, and cooperation among workgroup members. It largely contributes to safe performance.	[[99], [100], [101], [102], [103], [104], [105], [106]]
**Individual**	Self-efficiency (SE)	H10	Regarding safety, self-efficiency refers to employees' adequate competency and qualification to observe safety requirements. This competency motivates employees to control their coworkers' safety behavior.	[[107], [108], [109], [110], [111], [112], [113]]
	Knowledge and awareness (K&A)	H11	Knowledge and awareness about safety indicates employees' perception of hazards in the workplace.	[[114], [115], [116], [117], [118], [119], [120], [121], [122]]
	Safe behavior (SB)	H12	Employees' safe behavior refers to the risk-taking behaviors exhibited by employees and their compliance with organizational safety routines and regulations.	[[123], [124], [125], [126], [127], [128], [129], [130], [131]]

**Fig. 2:**
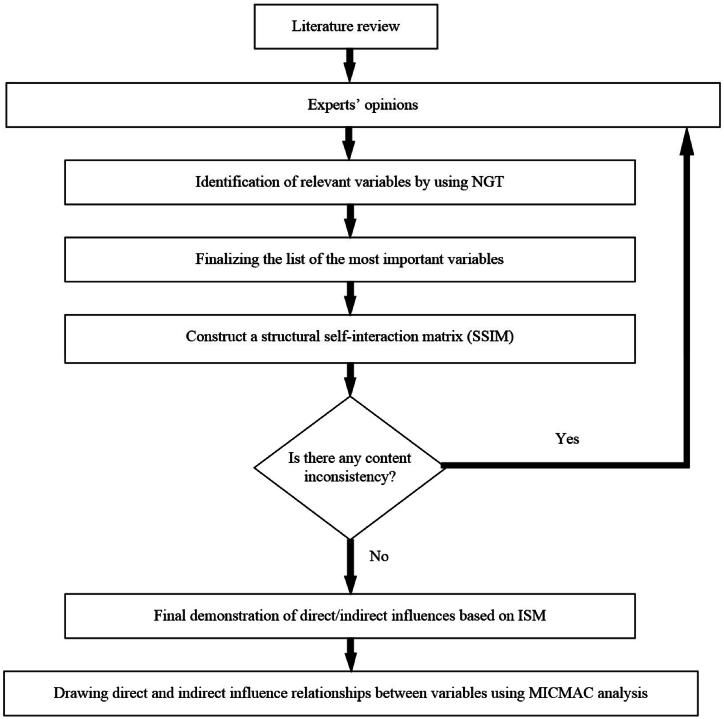
Flowchart of performing this research.

In this study, the ISM-MICMAC technique was used as an approach to finding a solution. Being used in various research works with different subjects, this method has become popular over time [132]. This implies the necessity and importance of using the ISM-MICMAC technique for BIM-based preventable risk identification and mitigation in mass housing projects, as is practiced in the present study. Fig. 2 presents the flowchart for performing this research.

### 2.2 Interpretive structural modeling (ISM)

Warfield first presented the ISM in 1974. This technique is a systematic application of the graph theory by which one can decompose complex systems into manageable components utilizing experts’ actual expertise and experiences [133,134]. Different stages of ISM are explained in the following.

Step 1 Forming a self-structured interpreted matrix (SSIM)

At this step, the variables are arranged in rows and columns and either of the following relationships is defined for different variable pairs [132]:

• V: When variable *i* affects variable *j*
• A: When variable *j* affects variable *i*
• X: When variables *i* and *j* affect one another mutually
• O: When variables *i* and *j* are not related

Step 2 Forming initial reachability matrix (IRM)

By converting different symbols of the SSIM into zeros and ones, one can obtain the IRM. The following rules shall be followed to prepare the IRM [132]:

• If (*i, j*) is represented by V in the SSIM, then (*i, j*) and (*j, i*) are converted to 1 and 0 in the IRM, respectively.
• If (*i, j*) is represented by A in the SSIM, then (*i, j*) and (*j, i*) are converted to 0 and 1 in the IRM, respectively.
• If (*i, j*) is represented by X in the SSIM, then both (*i, j*) and (*j, i*) are converted to 1 in the IRM.
• If (*i, j*) is represented by O in the SSIM, then both (*i, j*) and (*j, i*) are converted to 0 in the IRM.

Step 3 Forming final reachability matrix (FRM)

Considering the additive association of different elements, one needs to make the IRM consistent. For example, if Factor 1 leads to Factor 2 and then Factor 2 leads to Factor 3, then Factor 1 shall lead to Factor 3 as well, and if the reachability matrix cannot reflect this relationship, it must be corrected to replace the missed relationships. To this end, one must elevate the IRM to the power of *K* + *1* (*K* ≥ 1), in such a way that a stable state establishes ($M^{K}=M^{K+1}$). Albeit, the powering of the matrix shall be done via Boolean matrix multiplication. According to this multiplication scheme, 1 × 1 = 1 and 1 + 1 = 1. In this way, some of the zero elements turn into 1s, which are then denoted as 1∗ [132].

Step 4 Determining the level of variables

At this step, the FRM is used to obtain received and output collections for each variable. To determine the priority levels of different variables, one should determine the reachability (output) and preliminary (received) collections for each variable. In order to determine the output collection for each element, the corresponding row must be checked. Indeed, the number of 1s along this row indicates directed lines that leave that element. The received collection of a variable includes all elements of the system that end at that particular variable. Once finished with determining the preliminary and reachability collections and identifying common elements (subscription collection), one should proceed to evaluate the level of each variable (element). In the first table, the variable for which the reachability and subscription collections are identical was assigned to the highest level of hierarchy on the ISM. The next table was formed by omitting the variable(s) assigned to the highest level. Similar to the first table, the second table is devised to identify the second-level variable(s). This process was iterated until all variables were assigned to different levels [132].

Step 5 Visualizing the ISM

At this step, the ISM is plotted based on the determined levels and the FRM. Once finished with recognizing the relationships and levels of the variables, they can be visualized in the form of a model.

### 2.3 MICMAC analysis

The main purpose of the MICMAC technique is to analyze the driving power and independence power of the variables [135,136]. The MICMAC analysis is based on the influence and dependence of each variable, providing for further investigation of the scope of each variable. In this analysis, variables are classified under either of four categories, namely autonomous, dependent, linkage, and independent variables.

• Autonomous: these variables exhibit little dependence and guidability and, in many cases, detach from the system as they are only loosely linked to the system. Any change in these variables cannot make any significant change into the system.
• Dependent: these variables exhibit strong dependence coupled with weak guidability. They show little influence on the system but are highly influenced by the system.
• Independent: these variables exhibit weak dependence coupled with high guidability. In other words, these variables are characterized by high influence and low dependence.
• Linking: these variables exhibit strong dependence coupled with strong guidability. That is, these variables highly affect and are highly affected by the system so that an even small change in them tends to fundamentally change the system

## 3 Result and discussion

### 3.1 Implementation of ISM for the factors affecting preventable risk mitigation in mass housing projects based on BIM

Table 3 reports the most important preventable safety risk mitigation factors in mass housing projects, which were herein extracted via BIM by means of library studies and experts’ opinions. The identified factors in Table 3 were imported into the SSIM. Different steps of implementing the ISM technique are demonstrated in Table 4, Table 5, Table 6, Table 7, Table 8, Table 9, Table 10.

**Table 4:** The SSIM for the factors affecting preventable safety risk mitigation in mass housing projects based on BIM.

**S. no.**	**Variable**	**SB**	**K&A**	**SE**	**TW**	**SS**	**RpS**	**RwS**	**SMS**	**EE**	**MR**	**CI**	**MC**
**1**	**MC**	V	X	V	V	X	A	V	X	X	O	O	
**2**	**CI**	A	A	A	X	A	A	A	X	X	O		
**3**	**MR**	V	X	X	V	X	A	A	V	V			
**4**	**EE**	X	X	X	X	A	A	A	V				
**5**	**SMS**	X	X	X	X	A	X	A					
**6**	**RwS**	V	V	V	O	V	V						
**7**	**RpS**	A	A	A	O	O							
**8**	**SS**	V	O	V	O								
**9**	**TW**	X	X	X									
**10**	**SE**	V	A										
**11**	**K&A**	V											
**12**	**SB**												

Initial reachability matrix (IRM) is obtained by converting the SSIM to a binary matrix, where the elements on the primary diagonal are set to 1.

**Table 5:** The IRM for the factors affecting preventable safety risk mitigation in mass housing projects based on BIM.

**Variable**	**MC**	**CI**	**MR**	**EE**	**SMS**	**RwS**	**RpS**	**SS**	**TW**	**SE**	**K&A**	**SB**
**MC**	1	0	0	1	1	1	0	1	1	1	1	1
**CI**	0	1	0	1	1	0	0	0	1	0	0	0
**MR**	0	0	1	1	1	0	0	1	1	1	1	1
**EE**	1	1	0	1	1	0	0	0	1	1	1	1
**SMS**	1	1	0	0	1	0	1	0	1	1	1	1
**RwS**	0	1	1	1	1	1	1	1	0	1	1	1
**RpS**	1	1	1	1	1	0	1	0	0	0	0	0
**SS**	1	1	1	1	1	0	0	1	0	1	0	1
**TW**	0	1	0	1	1	0	0	0	1	1	1	1
**SE**	0	1	1	1	1	0	1	0	1	1	0	1
**K&A**	1	1	1	1	1	0	1	0	1	1	1	1
**SB**	0	1	0	1	1	0	1	0	1	0	0	1

**Table 6:** The FRM for the factors affecting preventable safety risk mitigation in mass housing projects based on BIM.

**Variable**	**MC**	**CI**	**MR**	**EE**	**SMS**	**RwS**	**RpS**	**SS**	**TW**	**SE**	**K&A**	**SB**	**Driving Power**
**MC**	1	1∗	1∗	1	1	1	1∗	1	1	1	1	1	12
**CI**	1∗	1	0	1	1	0	1∗	0	1	1∗	1∗	1∗	9
**MR**	1∗	1∗	1	1	1	0	1∗	1	1	1	1	1	11
**EE**	1	1∗	1∗	1	1	1∗	1∗	1∗	1	1	1	1	12
**SMS**	1	1	1∗	1∗	1	1∗	1	1∗	1	1	1	1	12
**RwS**	1∗	1	1	1	1	1	1	1	1∗	1	1	1	12
**RpS**	1	1	1	1	1	1∗	1	1∗	1∗	1∗	1∗	1∗	12
**SS**	1	1	1	1	1	1∗	1∗	1	1∗	1	1∗	1	12
**TW**	1∗	1∗	1∗	1	1	0	1∗	0	1	1	1	1	10
**SE**	1∗	1	1	1	1	0	1	1∗	1	1	1∗	1	11
**K&A**	1	1	1	1	1	1∗	1	1∗	1	1	1	1	12
**SB**	1∗	1∗	1∗	1	1	0	1	0	1	1∗	1∗	1	10
**Dependence Power**	12	12	11	12	12	7	12	9	12	12	12	12	135/135

**Table 7:** First step of iteration for partition levels of variables.

**S. no.**	**Reachability set**	**Antecedent set**	**Subscription collection**	**Level**
**1**	MC,CI,MR,EE,SMS,RwS,RpS,SS,TW,SE,K&A,SB	MC,CI,MR,EE,SMS,RwS,RpS,SS,TW,SE,K&A,SB	MC,CI,MR,EE,SMS,RwS,RpS,SS,TW,SE,K&A,SB	1st
**2**	MC,CI,EE,SMS,RpS,TW,SE,K&A,SB	MC,CI,MR,EE,SMS,RwS,RpS,SS,TW,SE,K&A,SB	MC,CI,EE,SMS,RpS,TW,SE,K&A,SB	1st
**3**	MC,CI,MR,EE,SMS,RpS,SS,TW,SE,K&A,SB	MC,MR,EE,SMS,RwS,RpS,SS,TW,SE,K&A,SB	MC,MR,EE,SMS,RpS,SS,TW,SE,K&A,SB	
**4**	MC,CI,MR,EE,SMS,RwS,RpS,SS,TW,SE,K&A,SB	MC,CI,MR,EE,SMS,RwS,RpS,SS,TW,SE,K&A,SB	MC,CI,MR,EE,SMS,RwS,RpS,SS,TW,SE,K&A,SB	1st
**5**	MC,CI,MR,EE,SMS,RwS,RpS,SS,TW,SE,K&A,SB	MC,CI,MR,EE,SMS,RwS,RpS,SS,TW,SE,K&A,SB	MC,CI,MR,EE,SMS,RwS,RpS,SS,TW,SE,K&A,SB	1st
**6**	MC,CI,MR,EE,SMS,RwS,RpS,SS,TW,SE,K&A,SB	MC,EE,SMS,RwS,RpS,SS,K&A	MC,EE,SMS,RwS,RpS,SS,K&A	
**7**	MC,CI,MR,EE,SMS,RwS,RpS,SS,TW,SE,K&A,SB	MC,CI,MR,EE,SMS,RwS,RpS,SS,TW,SE,K&A,SB	MC,CI,MR,EE,SMS,RwS,RpS,SS,TW,SE,K&A,SB	1st
**8**	MC,CI,MR,EE,SMS,RwS,RpS,SS,TW,SE,K&A,SB	MC,MR,EE,SMS,RwS,RpS,SS,SE,K&A	MC,MR,EE,SMS,RwS,RpS,SS,SE,K&A	
**9**	MC,CI,MR,EE,SMS,RpS,TW,SE,K&A,SB	MC,CI,MR,EE,SMS,RwS,RpS,SS,TW,SE,K&A,SB	MC,CI,MR,EE,SMS,RpS,TW,SE,K&A,SB	1st
**10**	MC,CI,MR,EE,SMS,RpS,SS,TW,SE,K&A,SB	MC,CI,MR,EE,SMS,RwS,RpS,SS,TW,SE,K&A,SB	MC,CI,MR,EE,SMS,RpS,SS,TW,SE,K&A,SB	1st
**11**	MC,CI,MR,EE,SMS,RwS,RpS,SS,TW,SE,K&A,SB	MC,CI,MR,EE,SMS,RwS,RpS,SS,TW,SE,K&A,SB	MC,CI,MR,EE,SMS,RwS,RpS,SS,TW,SE,K&A,SB	1st
**12**	MC,CI,MR,EE,SMS,RpS,TW,SE,K&A,SB	MC,CI,MR,EE,SMS,RwS,RpS,SS,TW,SE,K&A,SB	MC,CI,MR,EE,SMS,RpS,TW,SE,K&A,SB	1st

**Table 8:** Second step of iteration for partition levels of variables.

**S. no**.	**Reachability set**	**Antecedent set**	**Subscription collection**	**Level**
**3**	MR,SS	MR,RwS,SS	MR,SS	2nd
**6**	MR,RwS,SS	RwS,SS	RwS,SS	
**8**	MR,RwS,SS	MR,RwS,SS	MR,RwS,SS	2nd

**Table 9:** Third step of iteration for partition levels of variables.

**S. no**.	**Reachability set**	**Antecedent set**	**Subscription collection**	**Level**
**6**	RwS	RwS	RwS	3rd

**Table 10:** Final levels of variables.

S. no.	Level number	Variable
**1**	1st	MC
		CI
		MR
		EE
		SMS
		RpS
		TW
		SE
		K&A
		SB
**2**	2nd	MR
		SS
**3**	3rd	RwS

Structural self-interaction matrix (SSIM) is composed of the dimensions and indices of the study and their comparisons. This matrix was filled by the experts and elites in the field of the core process. The data was then compiled with the help of the inferential – structural modeling (ISM) to build the final SSIM. The logic of ISM is based on non-parametric methods and mode value in frequency tables. Table 4 presents the SSIM of the present research.

Once the binary IRM was developed, one should proceed to design a final reachability matrix (FRM). In an IRM, the secondary associations must be controlled. That is, if the secondary associations imply that a direct impact shall be considered but it is not the case in the matrix, one should modify the table in such a way to properly reflect the secondary association as well. Scientifically speaking, FRM is obtained by introducing the exchangeability to the associations among the variables.

To sum up, the final levels of the factors affecting preventable safety risk mitigation in mass housing projects based on BIM are presented in Table 10.

### 3.2 MICMAC analysis for the factors affecting preventable risk mitigation in mass housing projects based on BIM

**Fig. 3:**
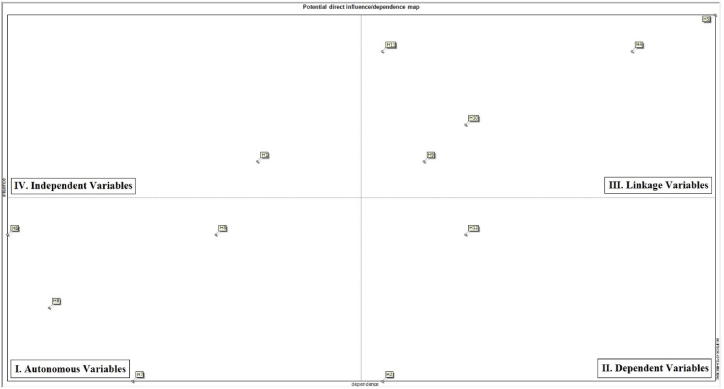
The potential direct influence/dependence map of variables.

**Fig. 4:**
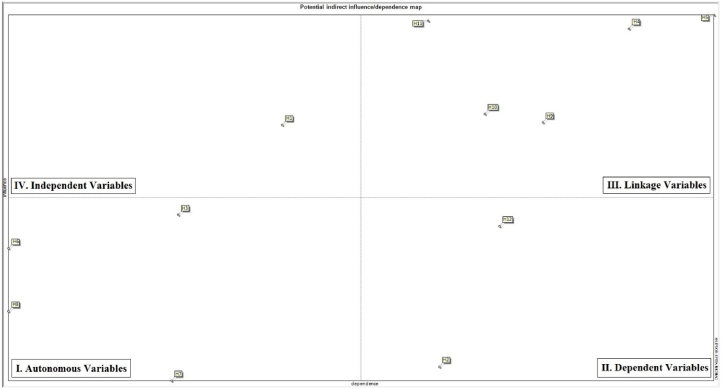
The potential indirect influence/dependence map of variables.

With the help of MICMAC software, analysis plots of variables in the direct influence matrix were used to investigate the output map of direct influences from the MICMAC software. Each array of the ISM can be plotted on a 2D domain. The ISM is indeed a symmetric matrix. The horizontal axis of the mentioned plot indicates the row-wise sum of scores for a particular variable, while the vertical axis refers to the column-wise sum of scores for that variable. Developed on the bottom-left of the plot, the output variables are not only weakly influential but also impose weak influences on other variables. Called autonomous variables, this group of variables imposes the smallest contributions to the system, so that one can simply eliminate them. The variables of the lowest influence coupled with the highest dependence (developed on the bottom right of the plot) are those that are most strongly affected by any change to the system conditions. These are referred to as dependent variables. The variables visualized on the top right of the plot not only impose the strongest influences on but also are most strongly affected by other variables, being denoted as linkage variables. In fact, these variables represent the instability of a system as they rapidly absorb the influences, due to their dependence on other variables, and rapidly transfer the influences, due to their strong influence on other variables. In other words, the high pace at which these variables can influence/be influenced by other variables tends to disturb the system's stability. Two classes of such variables have been identified, namely objectives (which we seek to achieve) and risk variables (which expose the system to danger). Independent variables are associated with the lowest dependence and highest influence (developed on the top left of the plot). These are effective drivers of the system. Fig. 3, Fig. 4 demonstrate potential direct and indirect influence/dependence maps of the variables, respectively.

According to the results of Fig. 3, Fig. 4 (direct and indirect influence maps), management commitment (H1) falls in the dependent matrix. That is, this variable exhibits low dependence coupled with high guidability, making it characterized by strong influence and weak dependence. The variables mutual relations (H3), reward system (H6), reporting system (H7), supervisors' supervision (H8) were found to be in the autonomous matrix. This category refers to independent variables that exhibit weak influence coupled with poor influence. The majority of the scenarios employees’ empowering (H4), safety management system (H5), teamwork (H9), self-efficiency (H10), knowledge and awareness (H11), however, ended up in the linkage matrix, which indicated their strong dependence coupled with high guidability. In other words, these latter variables are strongly influential and highly dependent, so any small change to them triggers fundamental changes into system. A couple of variables continuous improvement (H2) and safe behavior (H12), appeared in the dependent matrix, which demonstrated their strong dependence coupled with weak guidability. In general, such variables are highly dependent on the system but impose a weak influence on that.

**Fig. 5:**
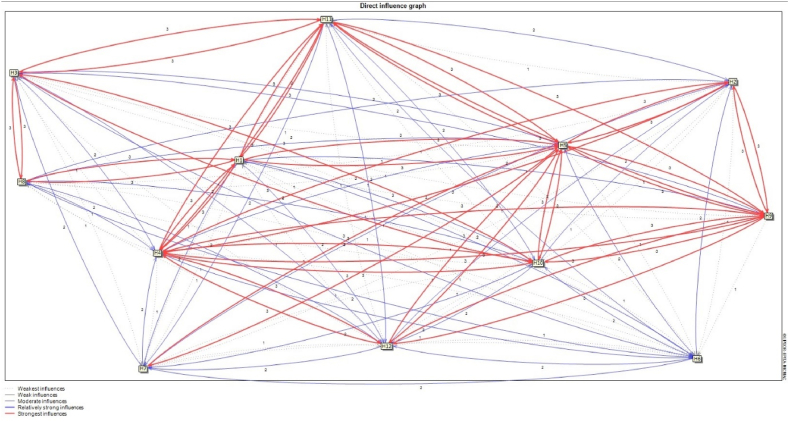
The direct influence relationship between the variables.

**Fig. 6:**
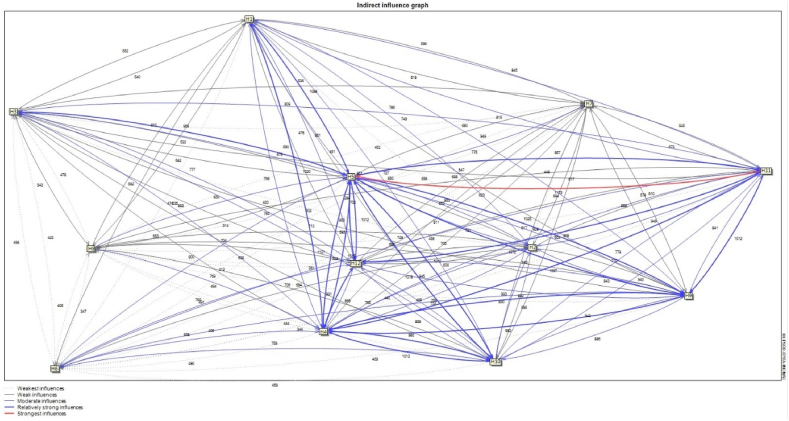
The indirect influence relationship between the variables.

Fig. 5, Fig. 6 visualize direct and indirect influence relationships between variables, respectively.

Fig. 5 shows the direct influence relationships between variables. According to this figure, direct influences were a combination of strong and weak relationships at 100 % level.

Fig. 6 illustrates the indirect influence relationships between variables. According to this figure, indirect influences among variables were reportedly strong.

**Fig. 7:**
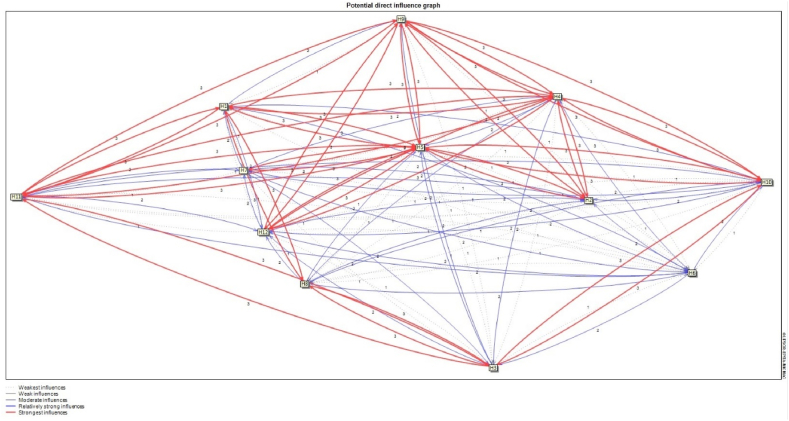
The indirect influence relationship between the variables.

**Fig. 8:**
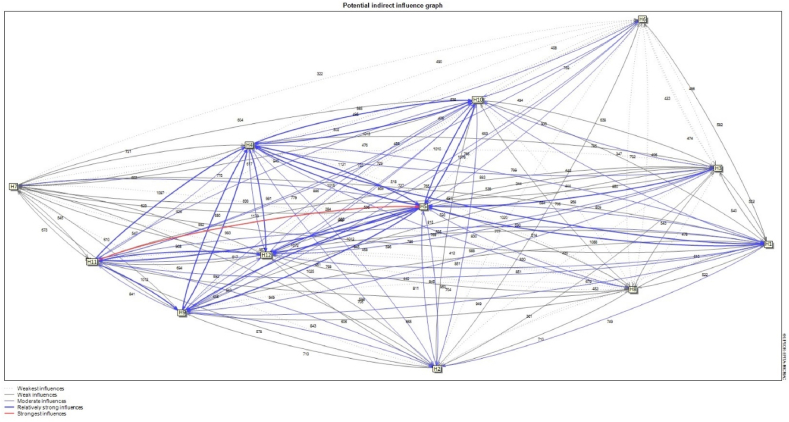
The potential indirect influence relationship between the variables.

**Fig. 9:**
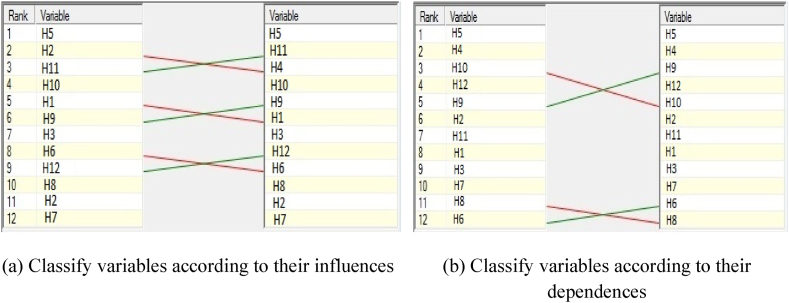
List of variables categorized by influence and dependence.

Fig. 7, Fig. 8 demonstrate the potential direct and indirect influence relationships between variables, respectively ( Fig. 9).

Finally, given that the X- and Y-axes of the direct influence matrix refer to the dependence and influence of the corresponding variable, respectively, the variables with the highest influence coupled with the weakest dependence are recognized as key drivers (the drivers are the most influential forces contributing to a particular subject and can direct the trend of mutual relationships between different components).

Iterations enable forecasting production, in which the requirement of fewer iterations (to achieve 100 % results) represents better conditions. Only two iterations were used in this case, the results of which can be found in Table 11.

**Table 11:** Matrix stability.

**Iteration**	**Influence**	**Dependence**
**1**	97 %	100 %
**2**	95 %	106 %

Through classifications, it is possible to examine the existing changes while forecasting the future conditions of each variable (both in terms of influence and dependence). The existing variables, arranged according to influence and dependence, can have different levels compared to each other.

An effective policy review requires knowledge of the single variables' priority levels. The ranking proportion, significantly contributing to the system evaluation, is represented through the proportion matrix in Table 12.

**Table 12:** Matrix proposition.

**Rank**	**Label**	**Direct influence**	**Label**	**Direct dependence**	**Label**	**Indirect influence**	**Label**	**Indirect dependence**	**Label**	**Potential direct influences**	**Label**	**Potential direct dependence**	**Label**	**Potential indirect influence**	**Label**	**Potential direct dependence**
**1**	H5	1037	H5	1203	H5	998	H5	1159	H5	1037	H5	1203	H5	998	H5	1159
**2**	H4	995	H4	1120	H11	992	H4	1079	H4	995	H4	1120	H11	992	H4	1079
**3**	H11	995	H10	954	H4	984	H9	999	H11	995	H10	954	H4	984	H9	999
**4**	H10	912	H12	954	H10	899	H12	958	H10	912	H12	954	H10	899	H12	958
**5**	H1	871	H9	912	H9	891	H10	945	H1	871	H9	912	H9	891	H10	945
**6**	H9	871	H2	871	H1	889	H2	902	H9	871	H2	871	H1	889	H2	902
**7**	H3	788	H11	871	H3	799	H11	892	H3	788	H11	871	H3	799	H11	892
**8**	H6	788	H1	746	H12	788	H1	755	H6	788	H1	746	H12	788	H1	755
**9**	H12	788	H3	705	H6	766	H3	657	H12	788	H3	705	H6	766	H3	657
**10**	H8	705	H7	622	H8	704	H7	651	H8	705	H7	622	H8	704	H7	651
**11**	H2	622	H8	539	H2	649	H6	499	H2	622	H8	539	H2	649	H6	499
**12**	H7	622	H6	497	H7	635	H8	498	H7	622	H6	497	H7	635	H8	498

The priority variables within the existing conditions, forecasting, and the potential presence of any actions in the system are presented in Table 12.

## 4 Conclusions

Failure to observe safety measures in construction projects represent a major cause of death and loss of national assets in any country. During the recent decades, applications of modern techniques (*e.g.*, BIM) in accident control and occupational health improvement have been introduced. This has led to the optimized safety of construction projects. In the present research, the identification and classification of different preventable risk mitigation factors in mass housing projects using the ISM-MICMAC technique following a BIM approach were investigated, and the most important criteria were identified and classified at different levels. Identification of these criteria and their importance contributes to the controllability and mitigation of accidents in mass housing projects.

The findings of the present research can be summarized as follows:

• Category 1: independent variables: this category includes the variables that exhibit the weakest dependence coupled with the strongest influence, making them stimulus variables. In this work, the management commitment (H1) is classified under this category, indicating the need for paying more attention to this variable.
• Category 2: dependent variables: this category includes the variables that exhibit the strongest dependence coupled with the weakest influence, making them susceptible to admit the largest effects from a change of conditions in a system. In this work, the continuous improvement (H2) and the safe behavior (H12) occupy the region corresponding to dependent variables.
• Category 3: autonomous variables: this category refers to independent variables that exhibit weak influence coupled with poor influence. Such variables are the least important ones in a system. They can be eliminated from the model though their effects may not be completely ignored. In this work, the mutual relations (H3), the reward system (H6), the reporting system (H7), and the supervisors' supervision (H8) were identified as autonomous variables.
• Category 4: linkage variables: this category refers to linkage variables that come with strong influence coupled with strong dependence. They impose the largest impacts on and are highly affected by other variables. Higher-level variables are affected by the linkage variables, which are, in turn, affected by the lower-level variables. Indeed, the linkage variables indicate the degree of instability in a system as they absorb effects rapidly, due to their dependence on other variables, and propagate them throughout the system thanks to their high influence. In this work, the employees' empowering (H4), the safety management system (H5), the teamwork (H9), the self-efficiency (H10), and the knowledge and awareness (H11) were identified as the linkage variables that fill in the gap between the safety and occupational accident reduction in the mass house construction projects.

According to the outputs of the present research, companies and practitioners in the field of mas house construction are recommended to focus on the management commitment (H1), the employees’ empowering (H4), the safety management system (H5), the teamwork (H9), the self-efficiency (H10), and the knowledge and awareness (H11) as the most significant variables that deserve the largest deals of attention. In other words, before proceeding to execute a mass house construction project, decision-makers shall formulate policies for mitigating the risks in such a project by focusing on the mentioned variables as the core factors.

The final results of the present research show that consideration of the identified and classified criteria in mass production projects can reduce work-related accidents in such projects – a finding that is in agreement with those of previous researchers [6,7,[11], [12], [13], [14], [15], [16], [17], [18], [19], [20], [21], [22], [23],[27], [28], [29],[37], [38], [39], [40], [41], [42], [43],[57], [58], [59], [60], [61], [62], [63], [64], [65], [66], [67], [68], [69], [70], [71], [72], [73], [74], [75]].

Limitations of the present research included the availability of participants (employees, project managers, and supervisors, as the elites contributing to decision-making on the topic of this research) for scoring the effective factors in technological applications of BIM for preventing safety hazards. Indeed, participants of such an activity for identifying, classifying, and ranking different factors of BIM application for safety hazard prevention in mass house construction projects must be elites possessing four critical characteristics: relevant knowledge and experience, enough tendency to participate, adequate time for participation, and efficient communication skills. On the other hand, it was infeasible to access every single member of the statistical populations, which implied that one should take time to identify relevant elites and collect their opinions. Given the importance of safety in civil projects, especially mass house construction, future researchers may use composite methods to identify and rank hazards in other fields (*e.g.*, road, dam, power plant construction projects, etc.) with specific work-related risks. The application of such novel methods as DANP (a hybrid of DEMATEL and ANP) or FCM (Fuzzy Cognitive Maps) and its combination with metaheuristic techniques (Learning Fuzzy Cognitive Map) can also serve as a topic for future studies. We further recommend more in-depth studies on safety management and its contribution to reduced construction accidents, which require a lot of experience and skill. Therefore, one should base such studies on opinions from knowledgeable experts who know the target systems very well to ensure the accuracy of the results. Timely and proper implementation of the proposed model and the formulated safety solutions, many of which were presented in this study, can draw other researchers’ attention to the importance and necessity of the presented model and motivate mass construction contractors toward observing the proposed solutions in an attempt to reduce work-related accidents in civil projects and mass house construction projects, in particular.

## Statement of data availability

The datasets used and/or analyzed during the current study available from the corresponding author on reasonable request.

## CRediT authorship contribution statement

**Amir Mohammad Maleki Toulabi:** Writing – review & editing, Writing – original draft, Software, Resources, Methodology, Formal analysis, Data curation, Conceptualization. **Towhid Pourrostam:** Validation, Supervision, Project administration, Methodology, Investigation, Conceptualization. **Babak Aminnejad:** Methodology, Data curation, Conceptualization.

## Declaration of competing interest

The authors declare that they have no known competing financial interests or personal relationships that could have appeared to influence the work reported in this paper.
